# Microdose Lithium Protects against Pancreatic Islet Destruction and Renal Impairment in Streptozotocin-Elicited Diabetes

**DOI:** 10.3390/antiox10010138

**Published:** 2021-01-19

**Authors:** Jiahui Zhang, Fnu Anshul, Deepak K. Malhotra, Juan Jaume, Lance D. Dworkin, Rujun Gong

**Affiliations:** 1 Division of Nephrology, College of Medicine, University of Toledo, Toledo, OH 43614, USA; Zhang.Jiahui@utoledo.edu (J.Z.); fnu.anshul@utoledo.edu (F.A.); deepak.malhotra@utoledo.edu (D.K.M.); lance.dworkin@utoledo.edu (L.D.D.); 2 Division of Endocrinology, College of Medicine, University of Toledo, Toledo, OH 43614, USA; juan.jaume@utoledo.edu

**Keywords:** type 1 diabetes mellitus (T1D), glycogen synthase kinase 3β (GSK3β), nuclear factor erythroid 2-related factor 2 (Nrf2), oxidative stress, microdose, lithium, therapy

## Abstract

Psychiatric use of lithium has been associated with hypoglycemic effects, but its effect on type 1 diabetes mellitus (T1D) is unknown. In streptozotocin (STZ) induced murine models of T1D, microdose lithium therapy improved hyperglycemia, attenuated body weight loss and prevented early signs of diabetic kidney injury. This beneficial effect was associated with preservation of pancreatic islet histology and β-cell production of insulin as well as mitigated oxidative damage of islets. Mechanistically, lithium in islets cells induced inhibitory phosphorylation of glycogen synthase kinase 3β (GSK3β), the major molecular target of lithium that has been recently implicated in non-canonical regulation of Nrf2 activity. In turn, Nrf2 antioxidant response was potentiated in islets, marked by nuclear translocation of Nrf2 and augmented expression of its target antioxidant enzyme heme oxygenase 1 (HO-1). Conversely, cotreatment with trigonelline, a selective blockade of Nrf2, offset the lithium enhanced Nrf2 antioxidant response in islets, blunted the protective effect of lithium on pancreatic islets and β-cells, and abolished the hypoglycemic activity of lithium in STZ-injured mice. Collectively, our findings suggest that microdose lithium confers a protective effect on islet β-cells via targeting the GSK3β-regulated Nrf2 antioxidant response and thereby ameliorates T1D and its related kidney impairment.

## 1. Introduction

Diabetes mellitus is among the top 10 causes of death in adults, and is a common, slowly progressing, serious and long-term health problem that is associated with multiple complications and requires timely and sustained treatment [1,2]. Diabetes imposes substantial medical and socioeconomic burdens to the global healthcare system [3]. It was estimated that approximately 4.2 million deaths are attributable to diabetes in 2019. In addition, direct diabetes-related health expenditure was estimated to be $760 billion globally in 2019, which undoubtedly had a negative impact on societies worldwide in the forms of higher medical costs, lost productivity, premature mortality and intangible costs from reduced quality of life [4]. Diabetes is classified into type 1 and type 2 diabetes (T1,2D). Type 1 diabetes mellitus (T1D), also known as juvenile-onset diabetes, is caused by T-cell-mediated autoimmune attack of pancreatic islet β-cells, leading to the islet failure to produce insulin [5,6,7]. Due to the absolute deficiency of insulin, T1D patients require lifelong supplement of exogenous insulin, which is inconvenient and is complicated by a myriad of side effects [8]. It is imperative to develop novel regimens to protect or even recover islet β-cells so that T1D patients may gain self-sufficiency in insulin production.

A number of natural products are known to act as insulin secretagogues and have antidiabetic or hypoglycemic activities [9]. In the past decades, much effort has been dedicated to translate these agents into clinical applications. Among these agents, lithium salts have attracted a lot of interest [10]. Since the late 1940s, lithium has been used as an effective therapy for bipolar psychiatric disorders [11]. During the use of lithium, it was unexpected to note that glucose tolerance was improved in some manic-melancholic patients [12]. Subsequently, it was demonstrated that lithium had an antidiabetic activity and improved glucose tolerance in patients with diabetes [13,14]. To this end, short-term lithium therapy was associated with an insulin-like effect and decreased blood glucose levels [15]. An early study by Rossetti also indicated that lithium ion is able to restore insulin sensitivity in rats with pancreatectomy-induced diabetes [16]. The exact molecular mechanism responsible for the insulin-mimetic activity of lithium is largely elusive. Conversely, discontinuation of lithium therapy in psychiatric patients has been associated with transient diabetes [17]. The molecular mechanism underlying this hypoglycemic or antidiabetic action of lithium is unclear. A couple of early studies demonstrated that lithium may have a direct effect on β-cells [18,19], however, the exact mechanism of action is not fully understood. In addition to the hypoglycemic effect, lithium has also been noted to exert a potent protective effect in a number of organ systems [20,21,22,23], including the kidney, liver, pancreas and brain. This beneficial effect has been attributed to the inhibition of glycogen synthase kinase (GSK) 3β, the major molecular target of lithium action [24,25]. As a ubiquitously expressed serine/threonine kinase that regulates numerous cellular pathways, GSK3β plays a key role in a number of cellular processes, including glycogen biosynthesis, immune response, carcinogenesis [26] and has been recently implicated in regulation of the nuclear factor erythroid 2-related factor 2 (Nrf2), a master regulator of antioxidant response, detoxification and also anti-inflammatory and other cytoprotective mechanisms [27,28]. Via targeting the GSK3β mediated regulation of Nrf2 antioxidant response, lithium has been shown to promote longevity and health span in *Drosophila* [29] and to protect against acute and chronic injuries in the kidney in animal models [30]. Oxidative stress is a key mechanism centrally involved in the pathogenesis of islet β-cells dysfunction and T1D [31]. It remains unknown if lithium is able to target the GSK3β mediated regulation of Nrf2 antioxidant response in islets and thereby protect against β-cell injury and T1D. This study is aimed to address this question by employing the streptozotocin-elicited murine models of β-cell injury and T1D.

## 2. Materials and Methods

### 2.1. Animal Experiment Design

Animal studies were carried out under the oversight of the Institutional Animal Care and Use Committee (IACUC) of the University of Toledo (108828), and conformed to the National Institutes of Health Guide for Laboratory Animals.

### 2.2. Murine Model of STZ-Induced Diabetes

Streptozotocin (STZ, Cayman Chemical Company, Ann Arbor, MI, USA) dissolved in sodium citrate (50 mM) buffer [32] or equal volumes of vehicle were injected into 10-week-old male C57BL/6 mice, which were fasted prior to the STZ injection. Mice were divided into three groups randomly (*n* = 5): (1) Control group: Mice received daily intraperitoneal (I.P.) injection of sodium citrate (50 mM) consecutively for 5 days. (2) STZ + LiCl group: Mice received a single subcutaneous (S.C.) injection of lithium chloride (LiCl) (40 mg/kg) and then were treated with daily I.P. injection of STZ (55 mg/kg/day) for 5 days. (3) STZ + NaCl group: Mice received a single subcutaneous (S.C.) injection of sodium chloride (NaCl), in a molar amount equal to lithium chloride given in group 2, and then were treated with daily I.P. injection of STZ (55 mg/kg/day) for 5 days. The body weight was checked every 3 days. On day 5, 8 and 12, blood samples were collected to measure the fasting blood glucose (FBG). All animals were sacrificed on day 12, and tissues, blood and urine were collected for subsequent experiments.

In a separate experiment, mice were randomized to the following treatment. (1) LiCl group: Mice received a single S.C. injection of lithium chloride (40 mg/kg) and then STZ (55 mg/kg/day) injections consecutively for 5 days. (2) NaCl group: Mice were treated with a single S.C. injection of sodium chloride in a molar amount equal to lithium chloride given in group 1, and then received STZ (55 mg/kg/day) injections consecutively for 5 days. (3) LiCl + Trig group: Mice were treated with a single S.C. injection of 40 mg/kg lithium chloride and an I.P. injection of the trigonelline (Trig) (1 mg/kg, Sigma-Aldrich, Saint Louis, MO, USA), a small molecule inhibitor of Nrf2 with high specificity [33], and then received STZ (55 mg/kg/day) injections consecutively for 5 days. On day 12, body weight was measured followed by a collection of tissues, blood and urine for further investigation.

### 2.3. Blood Glucose Level Measurements

Mice were starved for 6 h and blood samples were collected by the submandibular bleeding method. Blood glucose levels were measured using a glucometer and OneTouch Ultra Test Strips (Life Scan Europe, Inverness, UK).

### 2.4. Isolation and Ex Vivo Treatment of Mouse Islets

Mice were euthanized and islets were isolated by the standard collagenase digestion followed by the density gradient centrifugation method [34]. In brief, 3 mL of ice-cold collagenase solution (1.7 mg/mL, Sigma-Aldrich) was injected into the pancreas via the common bile duct. After dissection, the pancreas was incubated at 37 °C for 20 min, and islets were separated by density gradient centrifugation and then handpicked. Part of islets isolated from each animal group were precultured for 24 h in Roswell Park Memorial Institute (RPMI) medium 1640 containing 10 mM glucose and supplemented with 10% fetal bovine serum (FBS), 2 mM L-glutamine, 100U/mL penicillin and 100 μg/mL streptomycin at 37 °C. Islets were then washed, incubated in medium containing 1.4 mM glucose for 60 min, and afterwards transferred to medium containing 25 mM glucose at 37 °C. Insulin released in medium was measured 60 min later.

### 2.5. Isolation of Glomeruli

Glomerular isolation was carried out as previously described [35]. In brief, 5 mL of PBS containing 8 × 10^7^ Dynabeads M-450 (Dynal Biotech ASA, Oslo, Norway) was perfused into the kidney via the abdominal artery. Kidneys were cut into 1-mm^3^ pieces and digested in collagenase (1 mg/mL, Sigma-Aldrich) at 37 °C for 30 min. The tissue was then pressed gently through a 100-µM cell strainer (BD Falcon, Bedford, MA, USA), and glomeruli containing Dynabeads were gathered using a magnetic particle concentrator.

### 2.6. Urine Analyses

Urine samples were subjected to SDS-PAGE followed by Coomassie brilliant blue (Sigma-Aldrich) staining. Urine albumin and creatinine concentration were measured using a mouse albumin ELISA quantitation kit (Bethyl Laboratories Inc, Montgomery, TX, USA) and a creatinine assay kit (BioAssay Systems, Hayward, CA, USA).

### 2.7. Pancreas Morphology and Immunohistochemistry Analysis

Histology preparations were done with paraffin-embedded formalin-fixed pancreatic tissues, which were prepared into 3–5 μM sections and then subjected to hematoxylin–eosin (H&E) staining. Micrographs were taken at low magnification and then subjected to computerized morphometry with ImageJ software (NIH, Bethesda, MD, USA) to calculate total pancreas and islet areas, islet density and insulin-positive β-cells areas based on pixel analysis [36]. Peroxidase immunohistochemistry staining was performed using the primary antibodies for insulin (Cell Signaling Technology, Danvers, MA, USA), Nrf2 (Abcam, Cambridge, MA, USA), phosphorylation at serine 9 of GSK3β (p-GSK3β^(S9)^) (Cell Signaling Technology, Danvers, MA, USA), heme oxygenase 1 (HO-1), nitrotyrosine and 8-hydroxy-2′-deoxyguanosine (8-OHdG) (Santa Cruz Biotechnology, Santa Cruz, CA, USA) according to the manufacturer’s instructions.

### 2.8. Islet Immunofluorescence Analysis

Cryosections of the pancreas or kidney specimens were fixed, blocked and then labeled with primary antibodies against neutrophil gelatinase-associated lipocalin (NGAL), synaptopodin (SYNPO) (Santa Cruz Biotechnology, Santa Cruz, CA, USA), insulin (Cell Signaling Technology, Danvers, MA, USA), Nrf2 (Abcam, Cambridge, MA, USA), Ki67 or p-GSK3β^(S9)^ (Cell Signaling Technology, Danvers, MA, USA) and then stained with the Alexa Fluor-conjugated secondary antibodies (Invitrogen, Carlsbad, CA, USA). Finally, sections were counterstained with 4′,6-diamidino-2-phenylindole (DAPI) and visualized with a fluorescence microscope.

### 2.9. Western Immunoblot Analysis

Isolated pancreatic islets, isolated renal glomeruli and kidney tissues were homogenized in radioimmunoprecipitation (RIPA) buffer supplemented with protease inhibitors and protein samples were processed for immunoblot analysis [35]. Nuclear fractions were prepared by using the NE-PER kit (Thermo Scientific, Rockford, IL, USA). The antibody against Nrf2 was purchased from Abcam (Cambridge, MA, USA). The antibodies against insulin, cleaved caspase-3 and p-GSK3β^(S9)^ were purchased from Cell Signaling Technology (Danvers, MA, USA) and those against SYNPO, HO-1, GSK3β, NGAL, nitrotyrosine, histone and β-actin were purchased from Santa Cruz Biotechnology (Santa Cruz, CA, USA).

### 2.10. Statistical Analysis

For immunoblot analysis, bands were scanned and the integrated pixel density was determined using a densitometer and the ImageJ analysis program. The required animal numbers per group were calculated by power analysis to reliably detect meaningful effect size. All data were subjected to normality test by the Shapiro–Wilk normality test and proved to be normally distributed. All data were expressed as mean ± SD. Statistical analysis of the data from multiple groups was performed by a one-way ANOVA followed by the Tukey test. *p* < 0.05 was considered significant.

## 3. Results

### 3.1. A Single Injection of Microdose Lithium Protects against STZ-Elicited Diabetes and Attenuates Early Signs of Diabetic Kidney Injury in Mice

**Figure 1:**
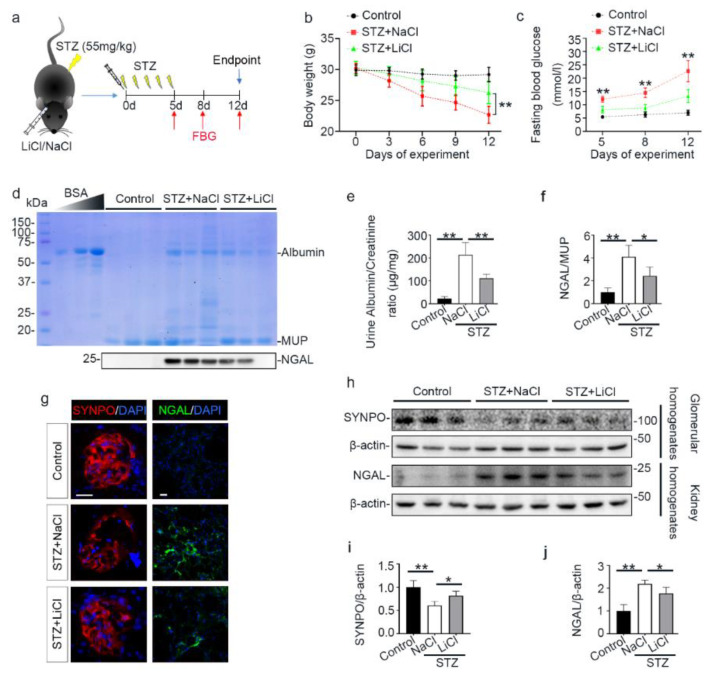
Microdose lithium protects against diabetes in STZ-injured mice and mitigates early signs of diabetic kidney disease. Mice received a single injection of lithium chloride or sodium chloride, and then were treated with a daily injection of STZ (55 mg/kg/day) or vehicle for 5 days. Fasting blood glucose was measured on indicated days. On day 12, mice were sacrificed. (**a**) Schematic diagram illustrates the animal experimental design. (**b**) Body weights were measured every 3 days; data were presented as mean ± SD; ** *p* < 0.01 on day 6, 9 and 12 (*n* = 5). (**c**) Fasting blood glucose levels were measured on indicated time points and data were presented as mean ± SD; ** *p* < 0.01 versus STZ + LiCl (*n* = 5). (**d**) Urine was collected on day 12 and urine samples (10 μL) were subjected to SDS-PAGE followed by Coomassie brilliant blue staining. Bovine serum albumin (BSA, 1 μg, 3 μg and 6 μg) served as a standard control. Lower panel: representative Western blot analysis of NGAL in urine samples. (**e**) The urine samples were processed for urine albumin ELISA assay adjusted for urine creatinine concentrations. Data were presented as mean ± SD; ** *p* < 0.01 (*n* = 5). (**f**) Graphic presentation shows densitometric analyses of NGAL in urine, presented as relative levels normalized to MUP levels. Data were expressed as fold changes over control and presented as mean ± SD; * *p* < 0.05, ** *p* < 0.01 (*n* = 5). (**g**) Kidney cryosections were prepared for fluorescent immunohistochemistry staining for SYNPO (red) or NGAL (green) with DAPI (blue) counterstaining for nuclei. The loss of podocyte homeostatic proteins SYNPO in glomeruli was attenuated, and the expressions of tubules injury marker NGAL were mitigated in kidneys from STZ-injured mice after lithium treatment. Scale bar = 20 μm. (**h**) Glomeruli were isolated from the excised kidneys by the magnetic beads-based approach and prepared for immunoblot analysis for SYNPO and β-actin. Whole kidney homogenates were prepared for immunoblot analysis for NGAL and β-actin. (**i**) Graphic presentation shows densitometric analyses of SYNPO in renal glomeruli and (**j**) NGAL in the kidney, presented as relative levels normalized to β-actin levels. Data were expressed as fold changes over control and presented as mean ± SD; * *p* < 0.05, ** *p* < 0.01 (*n* = 5). Abbreviations: DAPI, 4′,6-diamidino-2-phenylindole; FBG, fasting blood glucose; LiCl, lithium chloride; MUP, major urinary proteins; NaCl, sodium chloride; NGAL, neutrophil gelatinase-associated lipocalin; STZ, streptozotocin; SYNPO, synaptopodin.

A growing body of evidence suggests that lithium is able to protect against acute organ injury and has hypoglycemic activity [37,38]. To determine the effect of lithium on experimental T1D, mice were treated with microdose lithium chloride or sodium chloride and then received daily injection of low-dose STZ for 5 consecutive days (Figure 1a). As the FDA approved first-line therapy for bipolar affective disorder, lithium has a narrow therapeutic window. The commonly used dose of lithium in murine models of neurobiological disorders is between 120 and 160 mg/kg. The optimal dose of lithium for peripheral organs is unknown, but a single microdose (40 mg/kg) that is only one-third of the neurobiological dose has been shown to be sufficient to protect against injury in peripheral organs in mice, including the kidney, with no discernible adverse effects [39,40]. As such, the present study also adopted this microdose of lithium (40 mg/kg) in the STZ-injured mice. Following STZ injury, mice developed typical symptoms of diabetes, characterized by progressive reduction in body weight and increase in fasting blood glucose levels (Figure 1b,c). This was associated with early signs of diabetic kidney injury on day 12, marked by microalbuminuria that is determined by urinary protein electrophoresis and quantified by urinary albumin to creatinine ratios. In addition, urinary excretion of NGAL on day 12 was also significantly elevated, denoting potential injury to renal tubules (Figure 1d–f). This was further corroborated by fluorescent immunohistochemistry staining of kidney specimens and by immunoblot analysis of kidney homogenates for NGAL. Consistent with the finding of microalbuminuria, glomerular injury, reflected by a loss of podocyte homeostatic proteins like synaptopodin (SYNPO), was noted, as shown by fluorescent immunohistochemistry staining of kidney specimens and by immunoblot analysis of isolated glomeruli (Figure 1g–j). Lithium therapy significantly prevented body weight loss, hyperglycemia and urinary excretion of albumin and NGAL, preserved glomerular expression of SYNPO, and diminished renal expression of NGAL, suggesting a beneficial effect against the STZ injury.

### 3.2. Lithium Preserves the Histology of Pancreatic Islets and Retains β-Cells Production of Insulin in STZ-Injured Mice

**Figure 2:**
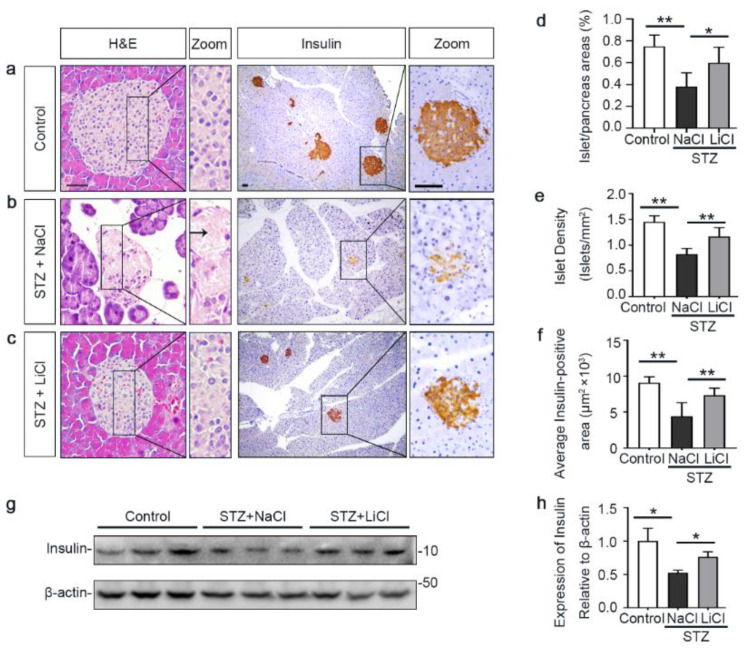
Lithium therapy preserves the histology of pancreatic islets and retains β-cell production of insulin in STZ-injured mice. Animals were treated as elaborated in Figure 1. (**a**–**c**) Representative micrographs demonstrate hematoxylin–eosin (H&E) staining of pancreas procured from animals on day 12 and peroxidase immunohistochemical staining for insulin. STZ injury induced apparent islet damages, characterized by pancreatic islet necrosis, as indicated by the arrow. These damages were significantly mitigated by lithium treatment. Scale bar = 50 µm. Computerized morphometric analysis determined (**d**) the percentage of islet/pancreas areas, (**e**) islet density and (**f**) average insulin-positive areas. Data were presented as mean ± SD; * *p* < 0.05, ** *p* < 0.01 (*n* = 5). (**g**) Pancreatic islets were isolated from the pancreas by collagenase digestion and density gradient centrifugation. Homogenates of isolated islets were prepared for immunoblot analysis for insulin and β-actin. (**h**) The expression levels of insulin were quantitated by densitometry and normalized with β-actin levels. Data were expressed as fold changes over control and presented as mean ± SD; * *p* < 0.05 (*n* = 5).

The underlying event of STZ-elicited T1D in mice is pancreatic islet injury and β-cells destruction. To determine if the above beneficial activity of lithium therapy stems from a possible effect on the islet injury, pancreatic specimens were procured and processed for histologic examination after hematoxylin–eosin (H&E) staining or peroxide immunohistochemistry staining for insulin, a marker for β-cells (Figure 2a–c). Shown in Figure 2b, STZ injury induced apparent islet damages, marked by diffuse pancreatic islet necrosis with sparing of the exocrine pancreatic acinar epithelium and ductal and connective tissues, shrinkage of the islet size and reduction in the number of islets. This was concomitant with β-cells destruction and disappearance, as evidenced by a substantial decrease of central islet immunoreactivity of insulin antigen. These signs of pancreatic islet injury and β-cells destruction were significantly mitigated by lithium treatment (Figure 2c). The morphologic findings were further quantified by computerized morphometric analysis, which consistently indicated a protective effect of lithium in terms of diverse parameters, including islet to pancreas area ratios, islet density and average insulin-positive areas (Figure 2d–f). Moreover, immunoblot analysis of the islet followed by densitometric analysis indicated that STZ injury considerably diminished insulin levels in islets and this effect was attenuated by lithium therapy (Figure 2g,h). In addition, the ex vivo experiments (Figure S1) indicated that islets isolated from lithium-treated STZ-injured mice exhibited an augmented insulin secretion in response to glucose stimulation as compared with islets from sodium-treated STZ-injured mice.

### 3.3. Lithium Therapy Attenuates STZ-Elicited Oxidative Stress in Pancreatic Islets, Concomitant with GSK3β Inhibition and Increased Nrf2 Expression

**Figure 3:**
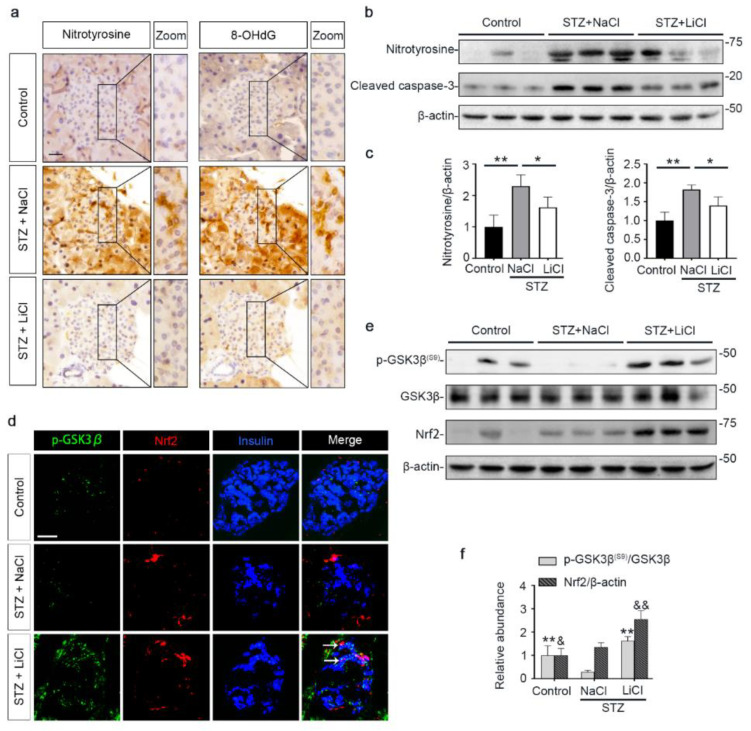
STZ-elicited oxidative stress in pancreatic islets is mitigated by microdose lithium therapy, concomitant with GSK3β inhibition and increased Nrf2 expression. Animals were treated as elaborated in Figure 1. (**a**) Formalin-fixed, paraffin-embedded pancreas specimens were sectioned and subjected to peroxidase immunohistochemistry staining for nitrotyrosine and 8-OHdG, and representative micrographs are shown. Scale bar = 20 µm. (**b**) Isolated islets were prepared for immunoblot analysis for nitrotyrosine, cleaved caspase-3 and β-actin, followed by (**c**) the densitometric analyses, in which the relative abundance of nitrotyrosine and cleaved caspase-3 were normalized with β-actin. Data were expressed as fold changes over control and presented as mean ± SD; * *p* < 0.05, ** *p* < 0.01 (*n* = 5). (**d**) Cryosections of pancreatic tissues were processed for fluorescent immunohistochemistry staining for p-GSK3β (green), Nrf2 (red) and insulin (blue). As compared with the STZ + NaCl group, the expressions of p-GSK3β, Nrf2 and the β-cell marker insulin were enhanced in the STZ + LiCl group. LiCl-treatment augmented the β-cell expression of p-GSK3β and Nrf2 in STZ-injured mice, as probed by colocalization of p-GSK3β and Nrf2 with insulin (white arrows). Scale bar = 50 μm. (**e**) Isolated islets were prepared for immunoblot analysis for indicated molecules. Representative immunoblots are shown. (**f**) Graphic presentation shows densitometric analyses of p-GSK3β^(S9)^ and Nrf2 in the pancreatic islets, presented as relative levels normalized to GSK3β and β-actin levels, respectively. Data were expressed as fold changes over control and presented as mean ± SD; ** *p* < 0.01 versus STZ + NaCl (*n* = 5); ^&^ *p* < 0.05, ^&&^ *p* < 0.01 versus STZ + NaCl (*n* = 5). Abbreviations: 8-OHdG, 8-hydroxy-2′-deoxyguanosine; GSK3β, glycogen synthase kinase 3β; Nrf2, nuclear factor erythroid 2-related factor 2; p-GSK3β, phosphorylated GSK3β at serine 9; p-GSK3β^(S9)^, phosphorylated GSK3β at serine 9.

Oxidative injury is a key mechanism underlying islet injury and β-cell dysfunction and contributes to the pathogenesis of T1D [41]. Upon STZ injury, oxidative stress was considerably elicited in pancreatic islets, marked by the amplified expression of nitrotyrosine and 8-hydroxy-2′-deoxyguanosine (8-OHdG), as shown by peroxidase immunohistochemistry staining and by immunoblot analysis of isolated islets followed by densitometric analysis (Figure 3a–c). This was associated with increased β-cell apoptosis in islets, as evidenced by immunoblot analysis of isolated islets for the apoptosis-related factor cleaved caspase-3 followed by densitometric analysis (Figure 3b,c). As the master regulator of the antioxidant response, Nrf2 expression in islets was apparently induced for self-defense, as revealed by fluorescent immunohistochemistry staining and by immunoblot analysis followed by densitometry (Figure 3d–f). GSK3β has emerged as the convergent point of a myriad of signaling pathways to regulate Nrf2 activity. GSK3β is constitutively active, while phosphorylation at serine 9 of GSK3β (p-GSK3β^(S9)^) is instrumental for its inhibition, ubiquitination and degradation. Therefore, the p-GSK3β^(S9)^ is a standard marker of GSK3β inhibition. Oxidative damages and apoptosis in islets were associated with reduced islet expression of p-GSK3β^(S9)^, denoting GSK3β hyperactivity. Consistent with the role as a standard inhibitor of GSK3β, lithium effectively counteracted GSK3β hyperactivity in islets of STZ-injured mice, as evidenced by increased expression of p-GSK3β^(S9)^. This was paralleled by more Nrf2 induction, resulting in significant attenuation of oxidative injury and apoptosis in pancreatic islets.

### 3.4. The Nrf2 Antioxidant Response to STZ Injury Is Reinforced in Pancreatic Islets by Lithium Therapy

**Figure 4:**
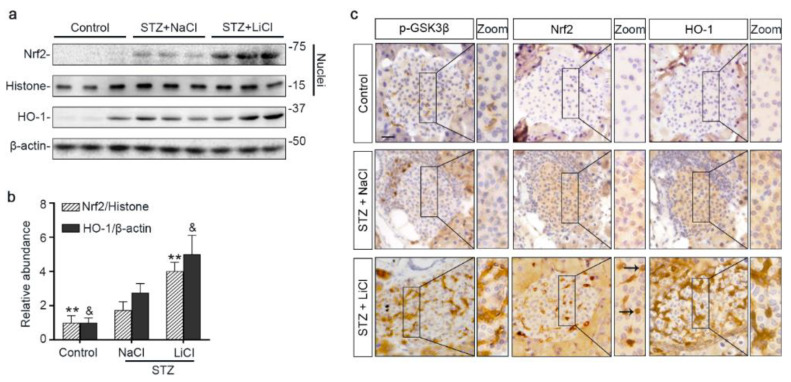
Microdose lithium therapy potentiates the Nrf2 antioxidant response to STZ injury in pancreatic islets. Animals were treated as elaborated in Figure 1. (**a**) Nuclear fractions of isolated islets were prepared, and together with whole islet homogenates, were processed for immunoblot analysis for indicated molecules. Representative immunoblots are shown. (**b**) Graphic presentation shows densitometric analyses of the abundance of Nrf2 in islet nuclei and the abundance of heme oxygenase (HO)-1 in islets, presented as relative levels normalized to histone and β-actin respectively. Data were expressed as fold changes over control and presented as mean ± SD; ** *p* < 0.01 versus STZ + NaCl; ^&^ *p* < 0.05 versus STZ + NaCl (*n* = 5). (**c**) Formalin-fixed, paraffin-embedded pancreas sections were subjected to peroxidase immunohistochemistry staining for p-GSK3β^(S9)^, Nrf2 and HO-1, and representative micrographs indicate that the Nrf2 antioxidant response was enhanced in islets and largely located to the nuclei in the STZ + LiCl group, as indicated by arrows. Scale bar = 20 µm. Abbreviations: HO-1, heme oxygenase 1; Nrf2, nuclear factor erythroid 2-related factor 2; p-GSK3β, phosphorylated GSK3β at serine 9.

To further ascertain if lithium-induced Nrf2 expression led to a reinforced antioxidant response, different fractions of isolated islets were subjected to immunoblot analysis followed by densitometry. Shown in Figure 4a,b, nuclear expression of Nrf2 was increased upon STZ injury, associated with a mild induction of HO-1, an Nrf2 target antioxidant enzyme, in the islet homogenates. Lithium treatment further enhanced nuclear expression of Nrf2 and promoted HO-1 production. In consistency, peroxidase immunohistochemistry staining of pancreas specimens indicated that lithium therapy potentiated the antioxidant response in islets to STZ injury, marked by an enhanced nuclear translocation of Nrf2 and reinforced HO-1 induction (Figure 4c). This effect coincided with induced expression of phosphorylated GSK3β in islets. In addition, in agreement with the role of Nrf2 in cellular proliferation and tissue repair/regeneration, the lithium-potentiated Nrf2 antioxidant response in β-cells in STZ-injured mice was concomitant with a reinforced β-cell regeneration, as evidenced by an increased amount of insulin-expressing β-cells positive for Ki67 per islet on fluorescence immunohistochemistry staining of pancreatic specimens (Figure S2).

### 3.5. Nrf2 Activity Is Required for the Lithium Reinforced Antioxidant Response to STZ Injury in Pancreatic Islets

**Figure 5:**
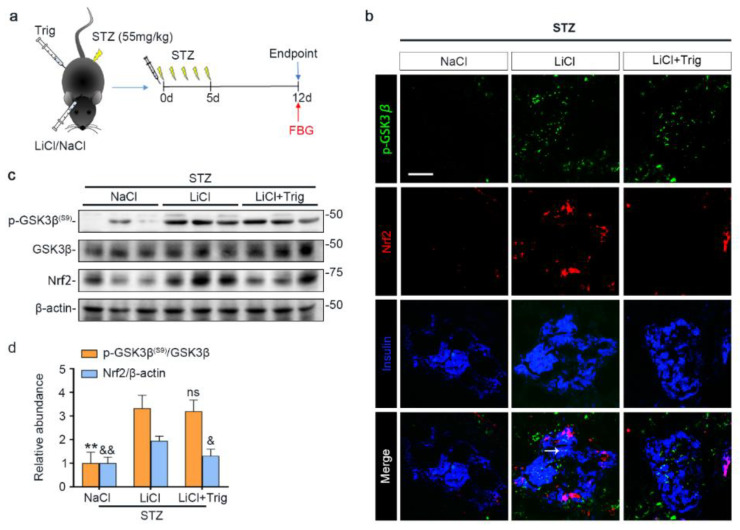
Trigonelline abrogates microdose lithium-reinforced Nrf2 activity in pancreatic islets in STZ-injured mice. Mice received a single injection of lithium chloride, sodium chloride or lithium chloride plus trigonelline (Trig), and then were treated with a daily injection of STZ (55 mg/kg/day) for 5 days. On day 12, fasting blood glucose (FBG) was measured and mice were sacrificed. (**a**) Schematic diagram depicts the animal study design. (**b**) Pancreas cryosections were prepared for fluorescent immunohistochemistry staining for p-GSK3β (green), Nrf2 (red) and insulin (blue). Despite a comparable expression of p-GSK3β in the LiCl group and the LiCl + Trig group, the lithium-promoted Nrf2 expression in β-cells was abolished by Trig treatment, as probed by colocalization of Nrf2 with the β-cell marker insulin (white arrow). Scale bar = 50 μm. (**c**) Representative Western blot analysis of pancreatic islet homogenates for indicated molecules. (**d**) Densitometric quantification of the abundance of p-GSK3β^(S9)^ and Nrf2 in islets, presented as relative levels normalized to GSK3β and β-actin respectively. Data were expressed as fold changes over NaCl group and presented as mean ± SD; ** *p* < 0.01 versus LiCl; ns: not significant versus LiCl; ^&^ *p* < 0.05, ^&&^ *p* < 0.01 versus LiCl (*n* = 5). Abbreviations: GSK3β, glycogen synthase kinase 3β; Nrf2, nuclear factor erythroid 2-related factor 2; p-GSK3β, phosphorylated GSK3β at serine 9; p-GSK3β^(S9)^, phosphorylated GSK3β at serine 9.

Lithium is known to affect a myriad of cellular signaling pathways, although GSK3β has been identified to be a major molecular target. To determine if the lithium promoted Nrf2 activity subsequent to GSK3β inhibition plays a major role in mediating the beneficial effect of lithium in STZ-injured mice, a selective inhibitor of Nrf2, Trig [33], was given at the same time of lithium therapy (Figure 5a). Shown in Figure 5b–d, Trig cotreatment largely abrogated the lithium potentiated Nrf2 induction in islets in STZ-injured mice but barely affected the lithium induced inhibitory p-GSK3β^(S9)^, as revealed by fluorescent immunohistochemistry staining and by immunoblot analysis of isolated islets followed by densitometry.

**Figure 6:**
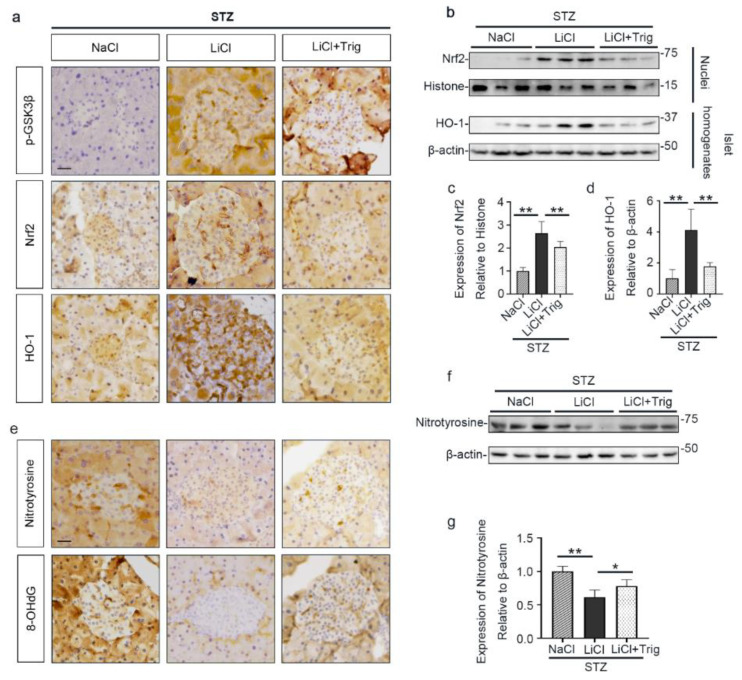
Nrf2 activity is required for the lithium reinforced antioxidant response to STZ injury in pancreatic islets. Animals were treated as elaborated in Figure 5. (**a**) Formalin-fixed, paraffin-embedded pancreas sections were processed for peroxidase immunohistochemical staining for p-GSK3β, Nrf2 and HO-1. Trig cotreatment barely affected the LiCl-induced p-GSK3β. Nuclear accumulation of Nrf2 and the expression of HO-1 in islet were augmented in the LiCl group, as compared with the NaCl group after STZ injury. This effect was significantly offset by Trig treatment. Scale bar = 20 µm. (**b**) Isolated pancreatic islets and their nuclear fractions were processed for immunoblot analysis for indicated molecules. (**c**) The abundance of Nrf2 in islet nuclei was determined by densitometric analysis of immunoblots as relative levels normalized to histone. Data were expressed as fold changes over NaCl group and presented as mean ± SD; ** *p* < 0.01 (*n* = 5). (**d**) The abundance of HO-1 in pancreatic islets was determined by densitometric analysis of immunoblots as relative levels normalized to β-actin. Data were expressed as fold changes over NaCl group and presented as mean ± SD; ** *p* < 0.01 (*n* = 5). (**e**) Formalin-fixed, paraffin-embedded pancreas sections were subjected to peroxidase immunohistochemical staining for nitrotyrosine and 8-OHdG. Scale bar = 20 µm. (**f**) Representative Western blot analysis of isolated pancreatic islets for indicated molecules. (**g**) The abundance of nitrotyrosine in pancreatic islets was determined by densitometric analysis of immunoblots as relative levels normalized to β-actin. Data were expressed as fold changes over NaCl group and presented as mean ± SD; * *p* < 0.05, ** *p* < 0.01 (*n* = 5). Abbreviations: 8-OHdG, 8-hydroxy-2′-deoxyguanosine; HO-1, heme oxygenase 1; Nrf2, nuclear factor erythroid 2-related factor 2; p-GSK3β, phosphorylated GSK3β at serine 9.

Moreover, the lithium reinforced Nrf2 antioxidant response, marked by nuclear translocation of Nrf2 and induced expression of HO-1 in pancreatic islets, was significantly offset by Trig, as shown by peroxidase immunohistochemistry staining and by immunoblot analysis of total homogenates or nuclear fractions of isolated islets followed by densitometry (Figure 6a–d). In parallel, the inhibitory effect of lithium on expression of oxidative markers, including nitrotyrosine and 8-OHdG, in islets was obviously abrogated by Trig cotreatment, as evidenced by peroxidase immunohistochemistry staining of pancreatic specimens and by immunoblot analysis of isolated islets followed by densitometry (Figure 6e–g). Collectively, blockade of Nrf2 counteracted the antioxidant effect of lithium therapy on STZ-injured islets, suggesting that Nrf2 activity is required for the lithium reinforced antioxidant response to STZ injury in pancreatic islets.

### 3.6. The Potentiated Nrf2 Activity Is Essential for the Beneficial Effect of Lithium Therapy on Islet Injury, β-Cell Destruction and T1D in STZ-Injured Mice

**Figure 7:**
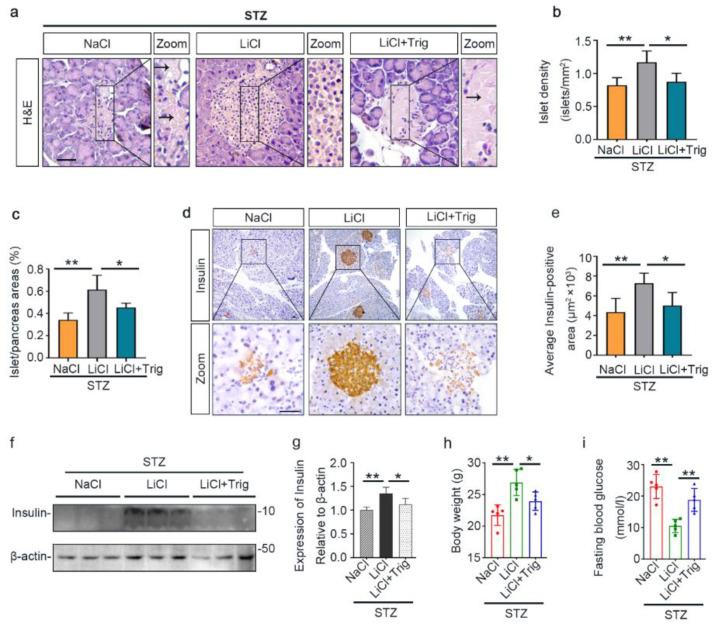
The potentiated Nrf2 activity is essential for the beneficial effect of microdose lithium therapy on islet injury, β-cell destruction and T1D in STZ-injured mice. Animals were treated as elaborated in Figure 5. (**a**) Formalin-fixed, paraffin-embedded pancreas sections were subjected to H&E staining for light microscopy. Representative microscopic images were shown. Islet damages were characterized by diffuse necrosis and were concomitant with β-cells destruction and disappearance (black arrows). Scale bar = 50 µm. (**b**) Islet number relative to pancreas areas and (**c**) islet areas relative to pancreas areas were measured by computerized morphometric analysis based on H&E staining of pancreatic sections. Data were presented as mean ± SD; * *p* < 0.05, ** *p* < 0.01 (*n* = 5). (**d**) Formalin-fixed, paraffin-embedded pancreas sections were prepared for peroxidase immunohistochemistry staining for insulin to demonstrate the protective effect of lithium on β-cells destruction, which was abolished by Trig treatment. Scale bar = 50 µm. (**e**) Average insulin-positive areas were measured by computerized morphometric analysis based on pancreatic sections stained for insulin. Data were presented as mean ± SD; * *p* < 0.05, ** *p* < 0.01 (*n* = 5). (**f**) Isolated pancreatic islets were subjected to immunoblot analysis for insulin and β-actin, respectively. (**g**) The abundance of insulin in pancreatic islets was determined by densitometric analysis of immunoblots as relative levels normalized to β-actin. Data were expressed as fold changes over the NaCl group and presented as mean ± SD; * *p* < 0.05, ** *p* < 0.01 (*n* = 5). (**h**) Body weights and (**i**) fasting blood glucose levels were measured on day 12. Data were presented as mean ± SD. * *p* < 0.05, ** *p* < 0.01 (*n* = 5).

In agreement with the counteractive effect of Trig on the lithium-enhanced antioxidant response, the protective effect of lithium on islet injury in STZ-injured mice was largely mitigated, as evidenced by diffuse pancreatic islet necrosis on H&E staining (Figure 7a). Likewise, the beneficial effect of lithium on islet density and islet to pancreas area ratios were offset after Trig treatment (Figure 7b,c). This was concomitant with a blunted lithium effect on β-cell destruction, as evidenced by substantial reduction in islet immunoreactivity of insulin antigen (Figure 7d). The morphologic findings were further quantified by computerized morphometric analysis (Figure 7e). Immunoblot analysis of isolated islets consistently demonstrated that Trig diminished the insulin levels that were retained by lithium therapy (Figure 7f,g). Consequently, the improving effects of lithium on body weight loss and hyperglycemia were largely abolished by Trig (Figure 7h,i), suggesting that Nrf2 plays a key role in mediating the beneficial effect of lithium therapy in STZ-elicited T1D.

## 4. Discussion

T1D is caused by autoimmune disorders and involves the destruction of pancreatic islets and β-cells, leading to an absolute deficiency of insulin and metabolic aberrations [42]. The etiology of β-cell injury is unclear, but oxidative stress is believed to play a key role [43]. Upon oxidative stress, an adaptive antioxidant response is harnessed by all tissues including the islet to sustain redox homeostasis and cellular integrity [44]. Central to this first line self-protective antioxidant mechanism is antioxidant enzymes and related molecules readily expressed in the stressed tissues under the control of Nrf2, including superoxide dismutase, catalases, HO-1 and glutathione peroxidases [45,46]. Unfortunately, the pancreatic islet is among the least endowed tissues in terms of expression and activity of intrinsic antioxidant enzymes [47]. Due to a lack of powerful antioxidant capacity, the islets might be the most vulnerable tissue to oxidative injury [48]. In addition, the hyperglycemic condition subsequent to the impaired β-cell production of insulin is known to elicit oxidative stress and cytotoxicity in the islet and other target organs of diabetes related damage, like the kidney [49], thus establishing a vicious cycle that perpetuates the progression of diabetes. The current mainstay of treatment for T1D is exclusively limited to insulin replacement therapy [50], which is costly, inconvenient but does not target the root cause of the disease. There is a great unmet need to develop novel therapy for T1D that targets islet β-cell injury. The present study demonstrates that microdose lithium is likely able to satisfy this need, protect the pancreatic islets against STZ injury and preserve the β-cell production of insulin, resulting in a mitigated T1D.

**Figure 8:**
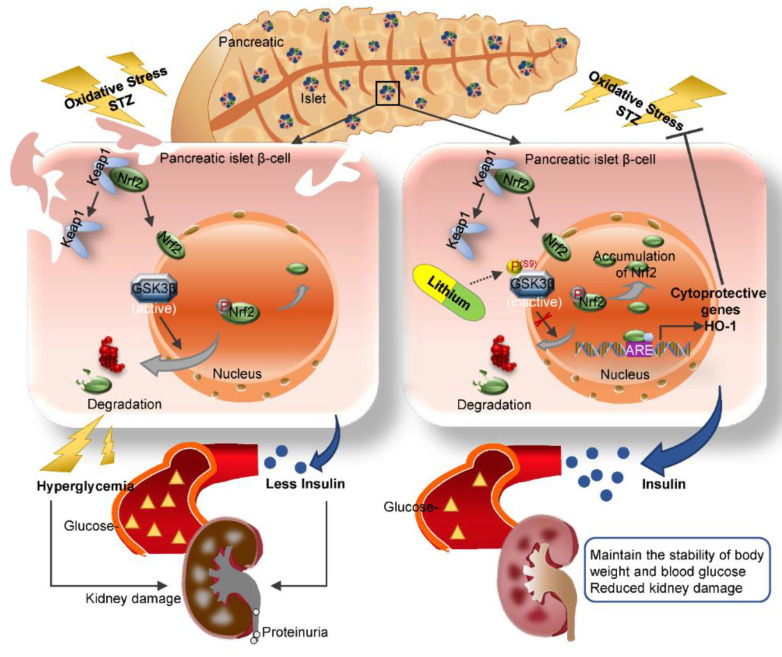
A schematic diagram depicts the protective effect of microdose lithium therapy on pancreatic islet destruction and renal impairment in streptozotocin-elicited diabetes. As a master regulator of the antioxidant response, Nrf2 is critical for sustaining redox homeostasis and cellular integrity. Under basal condition, Nrf2 is sequestered in the cytoplasm and associated with the actin anchored Kelch-like ECH-associated protein 1 (Keap1). Upon its activation triggered by oxidative stress, Nrf2 dissociates from Keap1 and subsequently translocates into the nucleus, where, Nrf2 recognizes and binds to a conserved antioxidant response element (ARE) and induces transcription of a battery of chemoprotective antioxidant genes, including those encoding antioxidant proteins like heme oxygenase (HO-1). Upon islet β-cell injury elicited by streptozotocin (STZ), oxidative stress is triggered and induces a spontaneous Nrf2 antioxidant response for self-defense, which is highly regulated by GSK3β, a key player in dictating Nrf2 nuclear exclusion and degradation and thereby in switching off the self-protective antioxidant stress response after injury. In response to injury, GSK3β is overactivated and thus Nrf2 antioxidant defense is greatly blunted, leading to β-cell necrosis, islet destruction, reduced production of insulin, hyperglycemia, diabetes and related complications like diabetic kidney impairment. This pathogenic process could be targeted by lithium, a standard inhibitor of GSK3β. Shown in this study in streptozotocin-induced murine models of T1D, microdose lithium therapy was able to induce inhibitory phosphorylation of GSK3β and reinforce the Nrf2 antioxidant response in β-cells, resulting in a protective effect on pancreatic islet destruction, diabetes and renal impairment.

Our finding is in line with a myriad of clinical observations that lithium has a hypoglycemic activity, the mechanism for which has long been elusive. Our data suggest that lithium is able to directly protect against oxidative injury in β-cells via potentiating an Nrf2 antioxidant response. The Nrf2 antioxidant response is the main mechanism for mammals to resist oxidative stress [27]. As the central to self-protective antioxidant mechanisms, Nrf2 upregulates wide-ranging genes encoding numerous antioxidants, detoxifying and cytoprotective enzymes and related molecules [51]. Nrf2 knockout significantly decreased the islet size in mouse models of β-cell-specific oxidative damage [52]. The Kelch-like ECH-associated protein 1 (Keap1) is an adaptor protein for Cullin3-based ubiquitin E3 ligase and negatively regulates Nrf2 [53]. Under basal conditions, Keap1 sequesters Nrf2 in the cytosol and facilitates its degradation in order to maintain it at low levels [54]. Under conditions of stress, Nrf2 dissociates from Keap1 and subsequently migrates to the nucleus. In the nucleus, Nrf2 recognizes and binds to a highly conserved antioxidant response element (ARE) and then induces transcription of a battery of antioxidant genes [51] that are responsible for quenching reactive oxygen and nitrogen species, increasing resistance to oxidative stress and recovering the cells to the basal state (Figure 8). In addition to the keap1-dependent regulation, the Nrf2 self-protective defense is also under the control of many other non-canonical regulatory pathways, which funnel to GSK3β as the convergent point [55]. To this end, it has been shown that Nrf2 is likely a cognate substrate for GSK3β, and that GSK3β overactivity promotes, whereas GSK3β inhibition intercepts Nrf2 phosphorylation and nuclear export [56]. In consistency, lithium therapy in our animals was able to mitigate GSK3β activity in the islets and reinforce the Nrf2 antioxidant response, marked by increased Nrf2 nuclear accumulation and elevated expression of the Nrf2 target antioxidant enzymes like HO-1, which are able to counteract the oxidative stress. This β-cell protective effect ultimately resulted in preservation of islet histology and production of insulin and ameliorated T1D, as evidenced by stabilized glycemia and body weight and lessened diabetic kidney injury and microalbuminuria (Figure 8).

As the FDA approved mood stabilizer, lithium has been safely used as the first line treatment for bipolar affective disorder for over 70 years [57,58]. Psychiatric use of lithium, however, requires a dose high enough to penetrate the blood brain barrier and to exert its mental activity, and thus has been associated with adverse effects to peripheral organs, such as the kidney, thyroid and other organs [59,60,61]. Recently, more and more studies indicated that the effective dose of lithium for peripheral organs is like much lower than the psychiatric dose. Indeed, the low dose of lithium used in this study is only 1/3 of the neurobiological dose of lithium used in murine models [39,40]. Yet, this low dose of lithium was sufficient to protect against β-cell injury and T1D and diabetic kidney injury [21,62]. Our finding may pave the way for prompt translation of the microdose lithium therapy to clinical use in patients with T1D. Admittedly, apart from GSK3β, lithium may affect numerous cellular signaling transducers [63]. However, there is evidence suggesting that GSK3β is likely the major, if not the only molecular target of lithium’s action [64]. In agreement, our study suggested that the potentiated Nrf2 response subsequent to lithium inhibition of GSK3β in islet β-cells is likely a major mechanism responsible for the beneficial effect of lithium in STZ-injured mice. In support of this, blockade of the Nrf2 activity by Trig largely abrogated the protective effect of lithium on STZ-elicited β-cell injury and the subsequent T1D.

However, our study is not with limitations. For instance, STZ-induced diabetes in rodents is oftentimes used in preclinical studies to model human T1D since it recapitulates loss of insulin-producing β-cells and basic pathophysiology in human T1D. However, human T1D is commonly caused by autoimmune disorders rather than chemical toxins. As such, the “chemical diabetes” elicited by STZ is pathogenically distinct from human T1D. In order to translate our findings to clinical applications, it is necessary to validate the salutary effect of lithium in additional models of autoimmune diabetes mellitus in future studies.

## 5. Conclusions

This study, for the first time, provided compelling evidence that targeting GSK3β by lithium is able to potentiate Nrf2 antioxidant response and mitigate the oxidative damage in pancreatic islets. Although in-depth studies and pilot trials are still warranted, our findings suggest that microdose lithium is likely a promising and pragmatic regimen for the treatment of T1D and its related complications.

## Data Availability

The data presented in this study are available on request from the corresponding author.

## Supplementary Materials

The following are available online at https://www.mdpi.com/2076-3921/10/1/138/s1, Figure S1: Islets isolated from lithium-treated diabetic mice exhibit an improved insulin secretion in response to ex vivo glucose stimulation. Animals were treated as elaborated in Figure 1. Islets were isolated from the differently treated animals via protocols specified in “Materials and Methods”. (a) Representative microscopic images of isolated islets. Scale bar = 50 μm. (b) An approximately equal amount of purified islets prepared from each group were cultured in high glucose medium (25mM) for 60 min. Conditioned medium was prepared for immunoblot analysis to detect the released insulin. Homogenates of cultured islets were subjected to immunoblot analysis for β-actin as a putative internal control. (c) Immunoblots were processed for densitometric analyses, in which the relative abundance of insulin was normalized with β-actin levels. Data were presented as mean ± SD; * *p* < 0.05 (*n* = 3–4). Figure S2: Lithium therapy promotes islet β-cell regeneration in STZ-injured mice. (a) Cryosections of pancreatic tissues were processed for fluorescent immunohistochemistry staining for insulin (green) and Ki67 (red) with 4′,6-diamidino-2-phenylindole (DAPI, blue) counterstaining for nuclei. Scale bar = 50 μm. (b) The numbers of insulin-expressing β-cells positive for Ki67 were quantified in each islet with 5 random islets per mouse. Data were presented as mean ± SD; ** *p* < 0.01 (*n* = 5).
