# Supplementary material for: Microdose Lithium Protects against Pancreatic Islet Destruction and Renal Impairment in Streptozotocin-Elicited Diabetes
Source: Antioxidants (Basel). 2021 Jan 19;10(1):138. doi: 10.3390/antiox10010138 (PMC7835906; doi:10.3390/antiox10010138)
Supplement: Supplementary file 1 [file antioxidants-10-00138-s001.pdf]

Supplementary Information:

Microdose lithium protects against pancreatic islet destruction and renal impairment in streptozotocin-elicited diabetes

Jiahui Zhang[1], Fnu Anshul[1], Deepak K. Malhotra[1], Juan Jaume[2], Lance D. Dworkin[1], Rujun Gong[1,*]

[1]Division of Nephrology and [2]Division of Endocrinology, University of Toledo College of Medicine, Toledo, Ohio, USA

*Corresponding author: Rujun.Gong@UToledo.edu

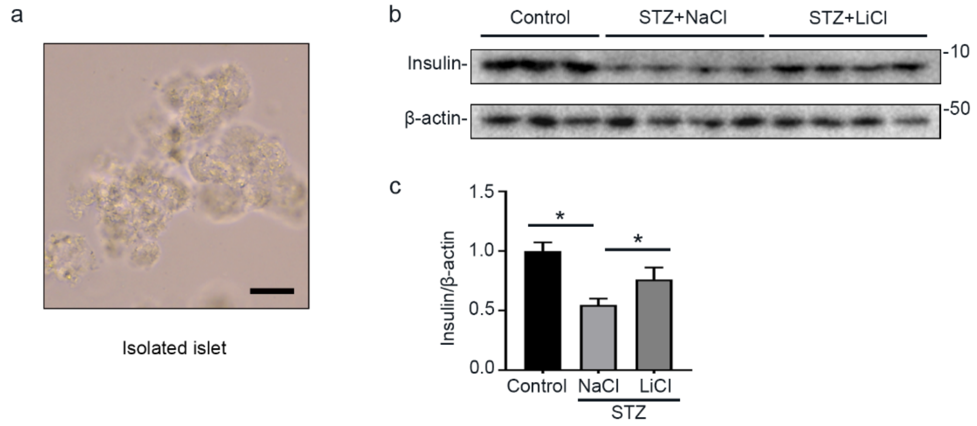

Supplementary Figure 1: Islets isolated from lithium-treated diabetic mice exhibit an improved insulin secretion in response to *ex vivo* glucose stimulation. Animals were treated as elaborated in Figure 1. Islets were isolated from the differently treated animals via protocols specified in "Materials and Methods". (**a**) Representative microscopic images of isolated islets. Scale bar = 50 μm. (**b**) An approximately equal amount of purified islets prepared from each group were cultured in high glucose medium (25mM) for 60 min. Conditioned medium was prepared for immunoblot analysis to detect the released insulin. Homogenates of cultured islets were subjected to immunoblot analysis for β-actin as a putative internal control. (**c**) Immunoblots were processed for densitometric analyses, in which the relative abundance of insulin was normalized with β-actin levels. Data were presented as mean ± SD; *$P < 0.05$ (n = 3–4).

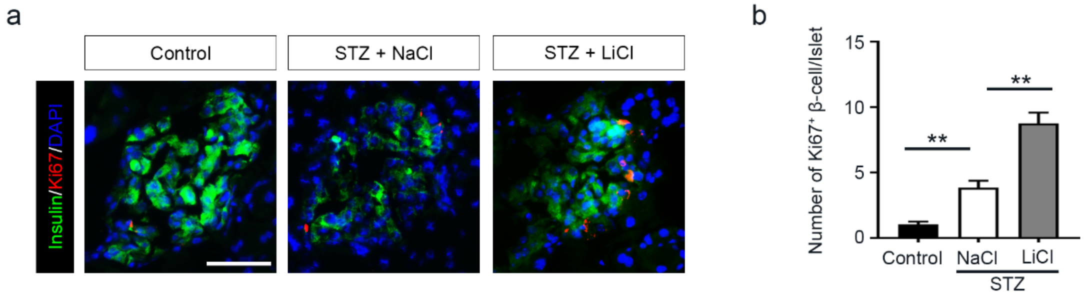

Supplementary Figure 2: Lithium therapy promotes islet β-cell regeneration in STZ-injured mice. (**a**) Cryosections of pancreatic tissues were processed for fluorescent immunohistochemistry staining for insulin (green) and Ki67 (red) with 4′,6-diamidino-2-phenylindole (DAPI, blue) counterstaining for nuclei. Scale bar = 50 μm. (**b**) The numbers of insulin-expressing β-cells positive for Ki67 were quantified in each islet with 5 random islets per mouse. Data were presented as mean ± SD; $^{**}P < 0.01$ ($n = 5$).
